# Altered gene expression and metabolism in fetal umbilical cord mesenchymal stem cells correspond with differences in 5-month-old infant adiposity gain

**DOI:** 10.1038/s41598-017-17588-4

**Published:** 2017-12-22

**Authors:** Peter R. Baker, Zachary W. Patinkin, Allison L. B. Shapiro, Becky A. de la Houssaye, Rachel C. Janssen, Lauren A. Vanderlinden, Dana Dabelea, Jacob E. Friedman

**Affiliations:** 1 Department of Pediatrics, Sections of Clinical Genetics and Metabolism, University of Colorado Anschutz Medical Campus, Aurora, CO 80045 USA; 2 Department of Pediatrics, Section of Nutrition, University of Colorado Anschutz Medical Campus, Aurora, CO 80045 USA; 3 Colorado School of Public Health, University of Colorado Anschutz Medical Campus, Aurora, CO 80045 USA; 4 Department of Pediatrics, Section of Neonatology, University of Colorado Anschutz Medical Campus, Aurora, CO 80045 USA

## Abstract

The intrauterine period is a critical time wherein developmental exposure can influence risk for chronic disease including childhood obesity. Using umbilical cord-derived mesenchymal stem cells (uMSC) from offspring born to normal-weight and obese mothers, we tested the hypothesis that changes in infant body composition over the first 5 months of life correspond with differences in cellular metabolism and transcriptomic profiles at birth. Higher long-chain acylcarnitine concentrations, lipid transport gene expression, and indicators of oxidative stress in uMSC-adipocytes were related to higher adiposity at 5 months of age. In uMSC-myocytes, lower amino acid concentrations and global differential gene expression for myocyte growth, amino acid biosynthesis, and oxidative stress were related to lower infant percent fat-free mass at 5 months of age, particularly in offspring of obese mothers. This is the first evidence of human infant adipocyte- or myocyte-related alterations in cellular metabolic pathways that correspond with increased adiposity and lower fat-free mass in early infancy. These pathways might reflect the effects of an adverse maternal metabolic environment on the fetal metabolome and genome. Our findings suggest that programmed differences in infant stem cell metabolism correspond with differences in body composition in early life, a known contributor to obesity risk.

## Introduction

Intrauterine development is a period of rapid growth and tissue remodeling, during which the fetus is particularly vulnerable to conditions that adversely impact organ development and disease propensity later in life. In addition to increased adiposity at birth, rapid postnatal weight gain in infants is a potent risk factor for childhood obesity and metabolic syndrome^1–3^, especially in infants born to obese mothers. Several mechanisms can explain these associations, including genetic predisposition to obesity, shared familial socioeconomic and behavioral factors, and specific intrauterine effects. However, one way in which prenatal exposures, particularly those resulting in fetal overnutrition, can increase offspring adiposity is via changes in adipose and skeletal muscle metabolism and fuel handling. Non-human primate models^4–6^ and limited human studies^7–10^ demonstrate that abnormalities in lipid handling and oxidative stress underlie risk in the offspring of obese, insulin resistant, and high-fat diet-consuming mothers. At the cellular level, obesity during pregnancy can accelerate fetal adipogenesis, suppress lipid metabolism, and impair energy sensing, affecting both adipocytes and skeletal myocytes^5,10,11^.

Although a broad range of cord blood metabolites are associated with newborn birth weight and adiposity in infants^12^, the hypothesis that cellular mechanisms underlying metabolic changes in newborn tissues impact early obesity risk in children remains largely untested. This is particularly due to the difficulties in tissue sampling of the human fetus and infant. To overcome this limitation, we have developed a novel human umbilical cord-derived mesenchymal stem cell (uMSC) model. We have demonstrated cell-specific differences in lipid handling and nutrient sensing related to maternal body mass index (BMI), maternal circulating lipids, and neonatal adiposity in differentiating uMSC-myocytes and -adipocytes^8–10^. However, whether cell-specific metabolomic and transcriptomic effects reflect longitudinal outcomes in infant adiposity gain is unknown. Phenotypic characteristics of these mesenchymal cells, taken at birth and removed from the infant environment, could provide insight into underlying mechanisms of infant fat accrual in the first months of life. Such cellular phenotypes might further inform general mechanisms of obesity risk in infants.

Here, we used our novel uMSC model to investigate underlying metabolic and gene expression differences related to maternal pre-pregnancy BMI (ppBMI) and changes in infant fat mass over the first 5 months of life. We tested the hypothesis that higher infant percent fat mass at 5 months of age corresponds with lipid handling and markers of cellular stress response in differentiating umbilical cord MSC taken at birth.

## Research Design and Methods

### Population

The Healthy Start Study is a longitudinal observational pre-birth cohort of 1,410 mother-infant pairs. Pregnant women were recruited at less than 24 weeks gestation through the University of Colorado Hospital obstetrics clinics during 2010–2014. Exclusion criteria included pre-existing type 1 or type 2 diabetes, a prior premature birth or fetal death, asthma with steroid management, serious psychiatric illness or a current multiple pregnancy. All women provided written informed consent and the study was approved by the Colorado Multiple Institutional Review Board. All experiments were performed in accordance with the relevant guidelines and regulations.

Mother and infant were followed with data collected including maternal ppBMI (obtained through medical record review and self-report). Serum samples for free fatty acids (FFA), triglycerides, glucose, and insulin were obtained at an average 27 weeks gestation. Neonatal body composition, determined by serial air displacement plethysmography (PEA POD, COSMED, Italy), was measured within 48 hours after delivery and at a median of 5 months of age. Percent fat mass (%FM), as measured by PEA POD, was calculated by dividing fat mass (g) by the total body mass. Percent fat-free mass (%FFM) is 100% minus %FM, and reflects lean body mass.

uMSCs were collected as part of the Healthy Start Baby Biology of Intrauterine Metabolic Programming (BUMP) project^8–10^. For these experiments, we utilized 24 uMSC samples from offspring of pre-pregnancy obese (OB; n = 12; BMI > 30 kg/m^2^) and normal-weight (NW; n = 12; BMI < 25 kg/m^2^) mothers matched for age, gestational age at delivery, and time to uMSC cell culture confluence. Maternal and infant phenotypes in the larger population have been previously described^13^. In the PEA POD analysis, all except one subject had %FM measures at birth and at 5 months of age; therefore, in our analyses, the total experimental group included 23 unique uMSC sets.

### uMSC growth and conditions

Our methods for growth and differentiation of uMSC have been described previously for myocytes and adipocytes^8,10^. Undifferentiated uMSC were grown to confluence and then exposed to lipid enriched myocyte or adipocyte differentiation media. Spent media and cell lysates were harvested on day 21 of differentiation. For further details regarding uMSC growth and media composition, please see Supplemental Methods.

### Media metabolomic analysis

Targeted metabolomic analysis was performed on spent media from all 23 cell sets, as previously described^10^, and included amino acid and acylcarnitine analyses. Amino acid analysis was accomplished using the Biochrom 30+ Amino Acid Analyzer (Cambridge, UK) through established protocols in an experienced clinical biochemical laboratory (Children’s Hospital Colorado). Acylcarnitine analysis was accomplished using a modified electrospray ionization MS/MS protocol^14^.

### RNA-Seq analysis

High throughput RNA-Seq analysis was performed on cell lysates using an Illumina High Seq. 2000 in a representative subset of samples (n = 5 for RPG, n = 8 for LPG, based on available cell lysate). Data was normalized and expression was quantified in fragments per kilobase of transcript per million mapped reads (FPKM) using Cufflinks. For further details regarding cell harvest, mapping, and quality control, please see Supplemental Methods.

### Statistical analysis

Quantitative amino acid, acylcarnitine, and gene-specific RNA-Seq data were analyzed using Prism 6.0 software (GraphPad, Cambridge, UK) with Student’s *t*-test analysis for each analyte and phenotypic outcome. False discovery rate (FDR) was determined using Benjamini-Hochberg procedure^15^, grouping analytes relative to known Kyoto Encyclopedia of Genes and Genomes (KEGG) database defined pathways or single enzyme complexes when possible. Significance in statistical analysis was given to nominal *P*-values of ≤0.05 with significant FDR ≤ 0.05, and marginal significance considered for FDR ≤ 0.15 (specified where applicable). STRING Database 10.0^16^ was used to analyze differential gene expression.

### Data availability

The datasets generated in the course of the current study are available from the corresponding author on reasonable request.

## Results

### Infant phenotypic analysis reveals two distinct groups characterized by adiposity at 5 months of life

**Figure 1:**
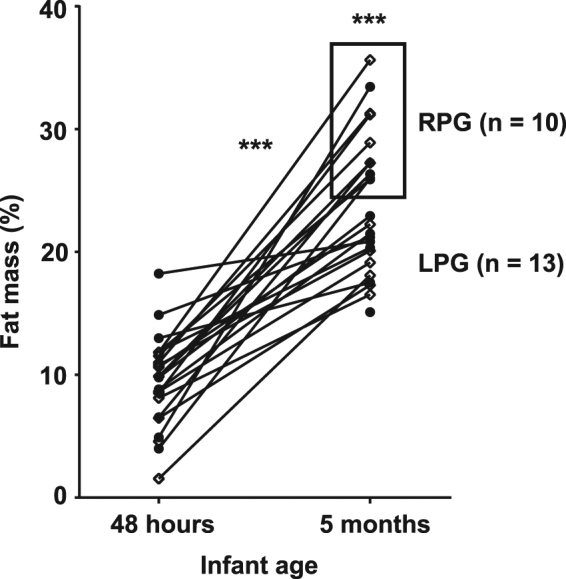
Percent fat mass (by PEA POD) measured at 48 hours (neonatal) and 5 months of life in offspring from normal-weight (◊) and obese (•) mothers. ***P < 0.001 for percent fat mass gain from neonate to 5 months of age and for rapid postnatal gain (RPG) in adiposity vs lower postnatal gain (LPG).

Our analysis focused on identifying characteristics of infants with rapid postnatal gain in %FM from both NW and OB mothers. The mother-infant pairs were initially selected for the Healthy Start BabyBUMP project based on maternal ppBMI. Figure 1 illustrates the infant PEA POD-determined %FM at birth and at 5 months of age. In our analysis of the whole group, we found the mean %FM at 5 months of age to be 24%, similar to the larger Healthy Start Study^13^. Relative to the mean %FM and %FFM in our experimental sample, there was a clear separation of offspring with higher 5 month-adiposity and higher postnatal gain in adiposity (RPG; higher %FM and lower %FFM at 5 months compared to the mean; n = 10) versus infants with lower postnatal gain in adiposity (LPG; lower %FM and higher %FFM at 5 months compared to the mean; n = 13). Differences between RPG and LPG in %FM at 5 months and %FM gain from birth to 5 months of age (%FM delta) were significant (both *P* < 0.001; Table 1). However, no differences were observed between the RPG and LPG groups in maternal ppBMI, gestational weight gain, maternal metabolic markers in the 2nd trimester (including FFA, triglycerides, glucose, and insulin), birth weight, infant gender, or exclusive breastfeeding (Table 1). Interestingly, no differences in infant weight gain (BM delta) between RPG and LPG groups were detected (Table 1), as well as no sex-related differences in phenotype including %FM at 5 months of age and %FM delta between the RPG and LPG groups (data not shown).

**Table 1:** Maternal and offspring phenotypes grouped by percent fat mass at 5 months of age.

**Group**	**Phenotype**	**RPG (n = 10)**	**LPG (n = 13)**	***P*** **-value (RPG/LPG)**
**Maternal**	ppBMI (kg/m^2^)	27.21 ± 6.48	30.34 ± 9.24	0.39 (0.9)
	GWG (kg)	9.9 ± 6.35	10.93 ± 6.28	0.71 (0.91)
	FFA (mg/dL)	446.5 ± 121.87	474.58 ± 160.45	0.67 (0.94)
	Glucose (mg/dL)	81.1 ± 10.85	78 ± 5.69	0.42 (1.04)
	Insulin (μU/mL)	14.4 ± 8.67	13.25 ± 5.82	0.73 (1.09)
	Triglycerides (mg/dL)	161.75 ± 37.98	147.1 ± 38.21	0.46 (1.1)
	HOMA-IR	3.09 ± 2.37	2.58 ± 1.26	0.55 (1.2)
	Gestational age (weeks)	39.63 ± 0.65	39.58 ± 0.85	0.89 (1)
	Age at delivery (y)	30.54 ± 5.24	28.33 ± 6.36	0.4 (1.08)
**Infant**	Sex, n (male/female)	5/5	9/4	0.42 (NA)
	Breastfed only, n	4/10	3/13	0.65 (NA)
	Birth weight (g)	3315 ± 256.55	3300.15 ± 405.87	0.92 (1)
	FM – Neo (%)	9.12 ± 2.75	10.35 ± 4.04	0.44 (0.88)
	FFM – Neo (%)	90.89 ± 2.75	89.65 ± 4.04	0.44 (1.01)
	FM – Neo (g)	0.29 ± 0.1	0.33 ± 0.15	0.46 (0.87)
	FFM – Neo (g)	2.85 ± 0.19	2.82 ± 0.31	0.79 (1.01)
	BM – Neo (g)	3.14 ± 0.23	3.15 ± 0.38	0.93 (1)
	BM (delta)	3.98 ± 0.59	3.58 ± 0.74	0.21 (1.11)
	%FM (delta)	20.33 ± 4	9.58 ± 3.71	4.43E-6* (2.12)
	Age, months (5mo visit)	4.42 ± 0.41	4.95 ± 0.77	0.07 (0.89)
	FM – 5mo (%)	29.44 ± 3.24	19.56 ± 2.29	5.83E-8* (1.5)
	FFM – 5mo (%)	70.56 ± 3.24	80.44 ± 2.29	5.83E-8* (0.88)
	FM – 5mo (g)	2.11 ± 0.35	1.33 ± 0.26	8.66E-6* (1.58)
	FFM – 5mo (g)	5.01 ± 0.29	5.46 ± 0.67	0.07 (0.92)
	BM – 5mo (g)	7.12 ± 0.52	6.8 ± 0.85	0.33 (1.05)

Data are mean ± SD. *P ≤ 0.001 by Student’s *t*-test.

RPG, rapid postnatal gain in adiposity; LPG, lower postnatal gain in adiposity; ppBMI, pre-pregnancy body mass index; GWG, gestational weight gain; FFA, free fatty acids; Neo, neonate; FM, fat mass; FFM, fat-free mass; BM, body mass; %FM, percent fat mass; 5mo, 5 months of age.

We further compared infant characteristics within the RPG and LPG groups by maternal ppBMI status (OB [BMI > 30 kg/m^2^] and NW [BMI < 25 kg/m^2^]; Supplemental Table 1). No differences were found among RPG and LPG infants who were born to NW mothers. Differences in maternal metabolic measures in the 2^nd^ trimester, gestational weight gain, birth weight, infant gender, or mode of feeding were not found between the RPG and LPG infants born to OB mothers.

### Higher adiposity at 5 months of age corresponded with higher long-chain acylcarnitines and lipid transport-related gene expression in uMSC-adipocytes

Based on differences in adiposity gain between RPG and LPG, we focused our uMSC analysis on these two groups using uMSC-adipocytes. We first analyzed acylcarnitines, amino acids, and gene expression differences between RPG vs LPG infants in the full experimental sample (n = 23). Additionally, we investigated the differences in long-chain acylcarnitines and lipid transport-related gene expression between RPG vs LPG by maternal ppBMI status, and in OB-only and NW-only groups.

**Figure 2:**
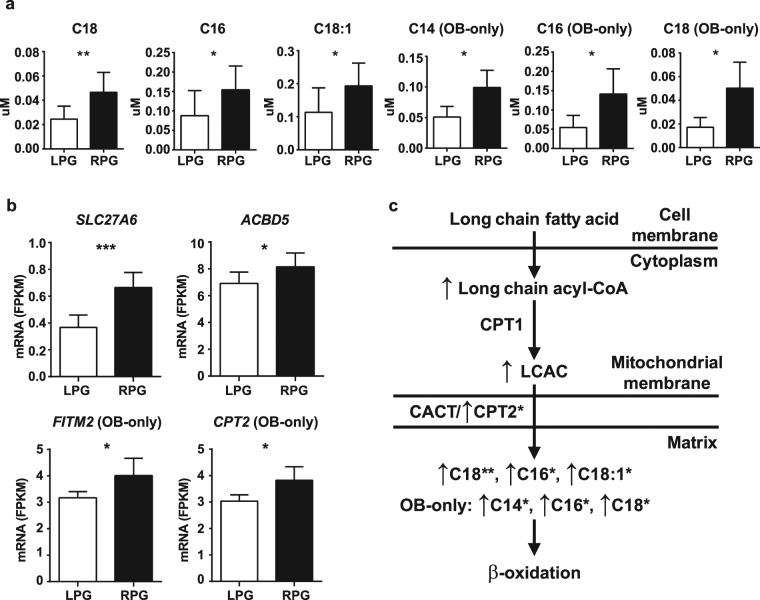
Acylcarnitine concentrations and targeted differences in gene expression in uMSC-adipocytes. (**a**) Long-chain acylcarnitine analysis in the lower postnatal gain (LPG) vs rapid postnatal gain (RPG) group in the total cohort and in the OB-only (infants from obese mothers only) cohort, as noted; n = 10 (RPG), n = 13 (LPG). (**b**) RNA-Seq data for differential gene expression in LPG vs RPG group in targeted genes involved in cellular lipid handling in total cohort or OB-only cohort, as noted; n = 5 (RPG), n = 8 (LPG). (**c**) Schematic of lipid transport showing higher expression of lipid transport genes in the RPG vs LPG group and higher concentrations of multiple long chain acylcarnitine species. LCAC, long-chain acylcarnitine; CACT, carnitine-acylcarnitine translocase. Acylcarnitines are specified using standard nomenclature, wherein long chain acylcarnitines are represented by C12-C18 species. Not pictured, medium chain acylcarnitines are represented by C6-C10, short chain by C2-C5, and free carnitine is C0. *P < 0.05, **P < 0.01, ***P < 0.001 by Student’s *t*-test. Metabolites FDR < 0.05 by Benjamini-Hochberg procedure.

Acylcarnitine analysis for uMSC-adipocytes revealed higher concentrations of the long-chain acylcarnitines C18 (*P* = 0.001), C16 (*P* = 0.03), C18:1 (*P* = 0.023), and the ratio C0/(C16 + C18) (*P* = 0.022) in the RPG vs LPG group (Fig. 2a; Supplemental Table 2). In the OB-only infants, saturated long-chain acylcarnitines including C14 (*P* = 0.02), C16 (*P* = 0.036), and C18 (*P* = 0.016) were higher for the RPG vs LPG group (Fig. 2a; Supplemental Table 2). No significant differences were found in the acylcarnitine analysis of NW-only infants (Supplemental Table 2).

RNA-Seq data in uMSC-adipocytes was analyzed for differential gene expression (RPG vs LPG) in targeted genes involved in cellular lipid handling. In the full experimental sample, higher expression of the membrane long-chain fatty acid transport protein 6 (*SLC27A6*; *P* < 0.001) and the peroxisomal long-chain lipid transporter acyl-CoA binding domain containing 5 (*ACBD5*; *P* = 0.04) was observed in RPG offspring compared with LPG offspring (Fig. 2b). In OB-only infants, there was higher expression in RPG offspring of both the membrane long-chain lipid transporter fat storage inducing transmembrane protein 2 (*FITM2*; *P* = 0.04) and the mitochondrial long-chain lipid transporter carnitine palmitoyltransferase 2 (*CPT2*; *P* = 0.03) compared with LPG offspring (Fig. 2b). Acylcarnitine and gene expression data are summarized contextually in the fatty acid transport and oxidation pathway schematic, Fig. 2c. No differences in lipid related gene expression between RPG and LPG offspring of NW mothers were observed (data not shown). Further, no other differentially regulated genes in fatty acid oxidation were noted, nor was there evidence of pathway enrichment by analysis of global differential gene expression, in uMSC-adipocytes. Together, these results support upregulated transport of fatty acids across the cell membrane, functional coupling for mitochondrial transport by CPT1 (to *create* long-chain acylcarnitines), and incomplete beta-oxidation in the RPG group compared with the LPG group, particularly in RPG offspring of OB mothers.

### In uMSC-adipocytes, amino acids and genes related to glutathione-mediated oxidative stress were different in RPG compared with LPG infants

**Figure 3:**
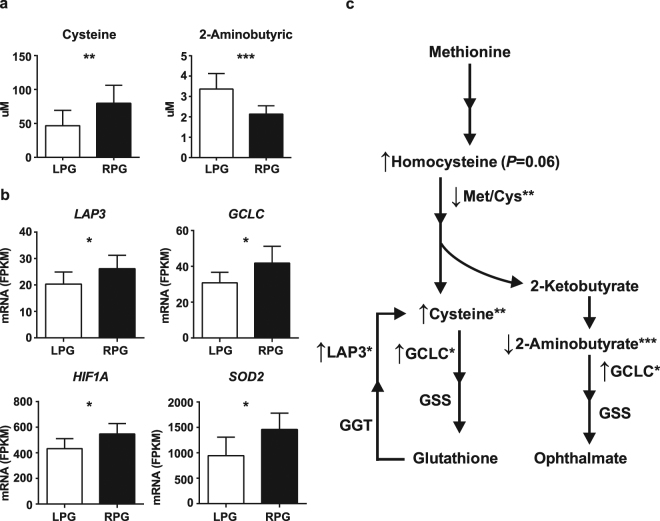
Differences in metabolites of the 1-carbon pathway and related differential gene expression in uMSC-adipocytes. (**a**) Metabolites of the 1-carbon pathway in rapid postnatal gain (RPG) group vs lower postnatal gain (LPG) group; n = 10 (RPG), n = 13 (LPG). (**b**) Differential gene expression (RPG vs LPG) in glutathione metabolism and oxidative stress-related genes; n = 5 (RPG), n = 8 (LPG). (**c**) Schematic of differences in 1-carbon analytes cysteine and 2-aminobutyrate accompanied by higher expression of glutathione-related genes in the RPG vs LPG group. GSS, glutathione synthetase; GGT, gamma-glutamyltransferase 1. *P < 0.05, **P < 0.01, ***P < 0.001 by Student’s *t*-test. Metabolites FDR < 0.05 by Benjamini-Hochberg procedure.

Amino acids in the 1-carbon metabolism pathway, specifically those related to glutathione metabolism, were found to be significantly different in the RPG vs LPG uMSC-adipocytes. Higher concentrations of cysteine (*P* = 0.007) and lower concentrations of 2-aminobutyrate (*P* = 0.0004) were observed in the RPG group (Fig. 3a), as well as differences in several related reaction ratios including Met:AABU (*P* = 0.0003), Met:Cys (*P* = 0.009), and Cys:AABU (*P* = 0.001) (Supplemental Table 2).

Both cysteine and 2-aminobutyrate are closely related to glutathione metabolism and oxidative stress. Targeted differential gene expression (RPG vs LPG) in related pathways revealed upregulation of leucine aminopeptidase 3 (*LAP3*; *P* = 0.05) and glutamate-cysteine ligase catalytic subunit (*GCLC*; *P* = 0.03), both directly related to glutathione recycling of cysteine, in the RPG group (Fig. 3b). *GCLC* also directly acts in the consumptive reaction of 2-aminobutyrate to form ophthalmic acid, a glutathione analog produced in the setting of increased glutathione recycling. Further analysis of genes related to oxidative stress revealed upregulation of hypoxia inducible factor 1 alpha (*HIF1A*; *P* = 0.03) and superoxide dismutase 2 (*SOD2*; *P* = 0.02) in the RPG offspring (Fig. 3b). Amino acid and gene expression data are summarized contextually in the glutathione/ophthalmic acid pathway schematic, Fig. 3c. Together, these data suggest increased cellular oxidative stress associated with RPG compared with LPG.

### Lower amino acid concentrations and global differential gene expression in growth, amino acid biosynthesis, and oxidative stress in uMSC-myocytes from RPG infants compared with LPG infants

**Figure 4:**
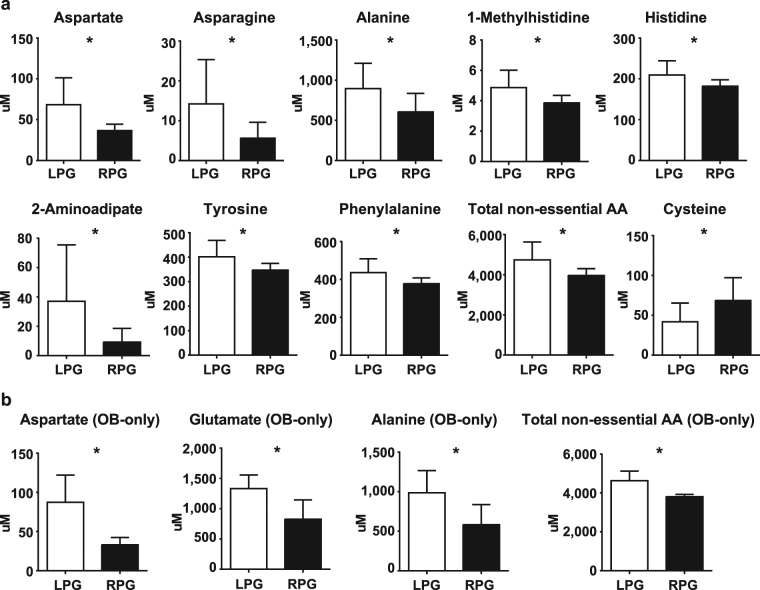
Amino acid analysis in uMSC-myocytes. (**a**) Amino acid analyses comparing offspring with rapid postnatal gain in adiposity (RPG) in the whole group and (**b**) in offspring from obese mothers (OB-only) vs infants with lower postnatal gain in adiposity (LPG). Cysteine was the only amino acid higher in the RPG group (**a**). n = 10 (RPG), n = 13 (LPG); *P < 0.05 by Student’s *t*-test. Metabolites FDR < 0.15 (marginally significant) by Benjamini-Hochberg procedure.

In uMSC-myocytes, multiple amino acids including aspartate (*P* = 0.01), asparagine (*P* = 0.04), alanine (*P* = 0.03), 1-methylhistidine (*P* = 0.024), histidine (*P* = 0.044), 2-aminoadipate (*P* = 0.05), tyrosine (*P* = 0.035), phenylalanine (*P* = 0.034), and total non-essential amino acids (*P* = 0.02) were lower in the RPG group (Fig. 4a; Supplemental Table 2), which had a significantly lower %FFM versus the LPG group. These were marginally significant by FDR. Specific to the OB-only group, aspartate (*P* = 0.016), glutamate (*P* = 0.017), alanine (*P* = 0.05), and total non-essential amino acids (*P* = 0.012) were lower in the RPG vs LPG offspring in uMSC-myocytes (Fig. 4b; Supplemental Table 2), while none were different between RPG and LPG in the offspring born to NW mothers (Supplemental Table 2). The only amino acid with higher concentrations in the RPG vs LPG group was cysteine (*P* = 0.028; Fig. 4a), similar to uMSC-adipocytes. Targeted differential gene expression (RPG vs LPG) for amino acid transporter and amino acid catabolism genes was not significant.

In testing global differential gene expression analysis in uMSC-myocytes, 1080 genes significantly differentially expressed between the RPG and LPG groups. Volcano plot and enrichment analysis through the STRING database are illustrated in Supplemental Figs. 1 and 2, respectively. GO Biologic Process enrichment was found for a total of 72 processes (Supplemental Table 3). Enrichment for upregulation of pathways related to myocyte development, but *downregulation* of pathways involved in Mitotic Cell Cycle (FDR = 1.5 × 10^−3^), Gene Expression, RNA Processing, and Cellular Biosynthetic Process (all FDR < 10^−7^) were observed in the RPG vs LPG group. KEGG pathway enrichment included downregulation of the Spliceosome pathway (FDR = 6 × 10^−4^) and GO RNA Processing (FDR = 2.8 × 10^−5^) (Supplemental Table 3). These downregulated indicators of uMSC-myocyte cell growth, as well as the aforementioned lower concentrations of multiple particularly non-essential (i.e. self-synthesized) amino acids, should be interpreted in the context of lower %FFM in the RPG vs LPG group.

In a sub-analysis, differential gene expression (RPG vs LPG) was analyzed in relation to maternal ppBMI (Supplemental Table 3). In the offspring of OB mothers, but not NW mothers, there was enrichment for upregulation of Apoptotic Process (FDR = 0.01) and Response to Unfolded Protein (FDR = 0.02), indicating cellular oxidative stress. This was accompanied by upregulation of Mitochondrial Organization, Oxidation-Reduction Process, and Organic Acid/Oxoacid Metabolic Process pathways (all FDR < 0.05). Enrichment for upregulation of similar KEGG pathways including Oxidative Phosphorylation and non-alcoholic fatty liver disease (NAFLD) (both FDR = 4 × 10^–3^) was also observed. Assessment of specific core enrichment genes in the NAFLD pathway (KEGG map04932) included upregulation of multiple Oxidative Phosphorylation genes associated with production of reactive oxygen species, as well as upregulation of adiponectin receptor *(ADIPOR1*; *P* = 0.02) and the interleukins *IL1A* (*P* = 0.008), *IL1B* (*P* = 0.005), and *IL8* (*P* = 0.03) (data not shown). These genes are all involved in lipotoxicity in the liver, but common to lipotoxicity in other tissues like skeletal muscle. Further, in offspring of OB mothers, but not NW mothers, there was enrichment for downregulation of the GO Biologic Process Gene Expression (FDR = 1.7 × 10^–22^) and Histone Modification (FDR = 1.2 × 10^–8^). The latter included core enrichment for lysine demethylase 4 A (*KDM4A*; *P* = 0.04), lysine demethylase 5 A (*KDM5A*; *P* = 0.004), histone deacetylase 1 (*HDAC1*; *P* = 0.03), and DNA methyltransferase 1 (*DNMT1*; *P* = 0.03), key genes in epigenetic modification (data not shown).

## Discussion

The cellular mechanisms for transgenerational propensity toward obesity, and the development of obesity pathophysiology over time in children, are poorly understood. Epidemiologic data indicate that exposures *in utero*, including maternal diet and BMI, as well as various exposures in the first few months of life, predispose some children to gain adiposity more readily thus establishing risk for obesity from an early age onward^13,17–19^. Established obesity, based on sampling of adolescent and adult adipocytes and myocytes, involves physiologic disturbances including increased lipid storage^20–22^, oxidative stress^23–25^, mitochondrial dysfunction^24–26^, and gain in fat mass over fat-free mass^27–30^. In this study, using uMSC from offspring with higher and more rapid gain in infant adiposity (RPG), we found evidence in both adipocyte and myocyte cell types that inherent differences in lipid and amino acid metabolism exist, as well as differences in markers of oxidative stress and altered mitochondrial gene expression compared with infants with lower adiposity and adiposity gain (LPG). Similar differences were found among RPG and LPG offspring born to OB mothers.

In uMSC-adipocytes, an overabundance of long-chain acylcarnitines was observed in the RPG group, and was more pronounced in offspring of OB mothers (Fig. 2c). Long-chain acylcarnitines (C12-C18) are known to be increased in the setting of established obesity^31–34^. They are biomarkers of incomplete beta-oxidation, and implicated as bioactive molecules that interfere with insulin sensitivity at the cellular level^22,31–34^. While we did not find evidence of dysregulated beta-oxidation at the level of gene expression, upregulation of multiple genes associated with lipid transport were affected. In particular, higher expression of *SLC27A6*, *FITM2*, and peroxisomal and mitochondrial transport-related genes were observed in the RPG group. *SLC27A6* is a ubiquitously expressed fatty acid transporter specific to long-chain fats^35^, has been linked to lipid handling in the setting of high dietary fat intake^36^, and is hypothesized to contribute to obesity-related pathophysiology although this has not been established^35,37^. *FITM2* is an evolutionarily conserved gene required for adipocyte lipid storage and lipid droplet accumulation^38^, and is affected directly by peroxisome proliferator-activated receptors, master regulators of lipid metabolism^39^. These data are suggestive of altered lipid partitioning, favoring storage over catabolic oxidation in RPG infants. Importantly, the infants with RPG did not gain excess total body weight relative to LPG infants. Rather, the increase in adiposity came at the expense of reduced fat-free mass, suggesting there might be cellular mechanisms that program nutrient partitioning that result in greater adipose gain over lean mass.

Through amino acid analysis, and analysis of related gene expression, we found evidence of differences in glutathione metabolism in the RPG versus the LPG groups in uMSC-adipocytes (Fig. 3c). Cysteine is a known biomarker of established obesity^40–42^, and is elevated in plasma and tissue related to oxidative stress and increased glutathione recycling^42^. We found upregulated glutathione-related gene expression. Particularly, *GCLC* (the first rate-limiting enzyme of glutathione synthesis) and *LAP3* (a peptidase that cleaves cysteine from glutathione), as well as key oxidative stress response genes *SOD2* and *HIF1A*, were upregulated in the RPG infants. Additionally, a lower concentration of 2-aminobutyrate was found in this group. This molecule is a byproduct of cysteine formation in the setting of glutathione homeostasis^43,44^, and is itself used in the formation of ophthalmic acid, a glutathione analog, in the setting of oxidative stress^45,46^. Ophthalmic acid is formed using the same rate limiting enzyme as glutathione (*GCLC*), in a reaction that consumes 2-aminobutyrate. So, whereas increased cysteine is a known indicator of glutathione recycling, reduced 2-aminobutyrate is also an indirect indicator of increased glutathione use^46^. These findings suggest that adipocytes from RPG infants are under increased oxidative stress and, together with increased lipid uptake and higher long-chain acylcarnitines, could contribute to the rapid increase in adiposity in the first 5 months of life observed in the RPG infants.

Reduced fat-free mass and increased fat mass without a change in birth weight has been reported in newborns of obese and gestational diabetic mothers^47,48^, and is a known risk factor of childhood obesity^3^. In uMSC-myocytes, lower amino acid concentrations were observed in a variety of different amino acid metabolism pathways related to lower percent fat-free mass. Interestingly, the only significantly higher amino acid in uMSC-myocytes was cysteine, as was found in uMSC-adipocytes. By global differential gene expression analysis, we found evidence of attenuated amino acid biosynthesis and related differences in gene expression for mitosis, DNA transcription, and RNA processing indicating attenuated cell growth and global synthetic dysfunction in uMSC-myocytes in relation to lower percent fat-free mass. Unique to the RPG offspring of obese mothers, evidence of mitochondrial and endoplasmic reticulum-associated oxidative stress response was found, as well as altered gene expression in pathways key in histone modification, implicating an epigenetic mechanism. Such alterations have been demonstrated in a variety of models in relation to maternal obesity and overnutrition^49^. In rat models, methylation changes to mitochondrial genes, and related mitochondrial dysfunction, were reported in fetal skeletal muscle taken from offspring of obese dams^50^. In human adult offspring of mothers with gestational diabetes, targeted differences have been shown in methylation of key metabolic regulatory genes including *PPARGC1A* ^51^. This is the first evidence that human myocyte-related cells can be programmed to maintain less fat-free mass, particularly in infants born to obese mothers.

Although our study was limited by a small sample size, potential confounders including gestational weight gain, *in utero* 2^nd^ trimester exposure to nutrients including FFA, triglycerides, glucose, and insulin, as well as infant covariates including birth weight, neonatal adiposity, gender, and exclusive breastfeeding were not significantly different between the RPG and LPG groups. It should be noted that in the much larger Healthy Start Study, factors including maternal ppBMI did influence adiposity gain by 5 months of age^13^. For this reason, and because our cohort was initially selected based on maternal ppBMI, we also stratified by maternal ppBMI in our uMSC analyses. The larger Healthy Start Study did find additional factors including gestational weight gain and exclusive breastfeeding that influenced adiposity at 5 months of age. Our analyses did not, which might be influenced by our small sample size or sample selection based on an equal number of obese and normal-weight mothers. While our uMSC model does not prove *in vivo* physiology, our results share similarities with fetal programming data in animal models, as well as novel patterns that are similar to humans with established obesity in later life. These results suggest the hypothesis that developmental changes in gene patterns and metabolic pathways present at birth can influence the offspring’s response to the environment in early postnatal life. While infant inborn, environmental, and maternal factors are likely important for changes in infant growth, these results support the idea that interventions, particularly during pregnancy, are needed to disrupt the cycle of transgenerational obesity.

## Electronic supplementary material

Supplemental Material
