# Supplementary material for: Altered gene expression and metabolism in fetal umbilical cord mesenchymal stem cells correspond with differences in 5-month-old infant adiposity gain
Source: Sci Rep. 2017 Dec 22;7:18095. doi: 10.1038/s41598-017-17588-4 (PMC5741772; doi:10.1038/s41598-017-17588-4)
Supplement: Supplementary file 1 — Supplemental Material [file 41598_2017_17588_MOESM1_ESM.pdf]

Altered gene expression and metabolism in fetal umbilical cord mesenchymal stem cells correspond with differences in 5-month-old infant adiposity gain

Peter R. Baker II[1], Zachary W. Patinkin[2], Allison L. B. Shapiro[3], Becky A. de la Houssaye[4], Rachel C. Janssen[4], Lauren A. Vanderlinden[3], Dana Dabelea[3] & Jacob E. Friedman[4]

Department of Pediatrics, Sections of Clinical Genetics and Metabolism[1] and Nutrition[2]; [3]Colorado School of Public Health; [4]Department of Pediatrics, Section of Neonatology, University of Colorado Anschutz Medical Campus, Aurora, CO 80045, USA

Correspondence and requests for materials should be addressed to P.B.
email: peter.baker@childrenscolorado.org
Phone: 303-724-2338
Fax: 720-777-7322
13123 E 16th Ave
University of Colorado Anschutz Medical Campus
Aurora, CO 80045

Supplemental Material

Methods

uMSC Growth and Conditions

Undifferentiated cells (Pass 2-3) from offspring of NW and OB mothers were thawed and grown to confluence in MSC growth media (MSC-GM; Lonza, Walkersville, MD). Cells were then sub-cultured into experimental plates. Once cells returned to confluence, they were separately exposed to myocyte differentiation media and adipocyte differentiation media. Information regarding stage of differentiation state can be found in Boyle et al. (2016)[1], as well as Janderová et al. (2003)[2] and Gang et al. (2004)[3] from which we based our protocols. The adipogenic differentiating uMSCs were induced for three cycles, with media changes every three days, and myogenic media was changed every 3 to 4 days. Media was harvested by pooling 3 x 500 uL aliquots (500 uL per well, 24 wells per plate) for analysis on day 21 of differentiation, following approximately 3 days continuous media exposure.

Media Metabolomic Analysis

Using the Biochrom 30+ Amino Acid Analyzer, 32 total amino acids were analyzed. Amino acid reaction ratios were calculated as the ratio of product over substrate of a known amino acid-associated reaction. Lower ratios corresponded to a more favorable reaction. Using MS/MS-ESI, 38 primary acylcarnitine species were analyzed, as well as calculated totals of short (C0-C5), medium (C6-C12), dicarboxylic acid (C3DC-C6DC), and long chain species (C14-C18) with and without hydroxyl groups. All analytes were placed in the context of known KEGG pathways. These included the central metabolic pathway for Ala/Asp/Glu (map00250), Val/Leu/Ile degradation (map00280), Tyrosine (map00350, which includes Phenylalanine), Cysteine, Methionine, Glycine, Serine, and Threonine (map00270 and 00260, considered 1-carbon metabolism), Arginine and Proline (map00330), Lysine (map00310), Tryptophan (map00380), and Histidine (map00340) metabolism. Acylcarnitines were associated with pathways of Fatty Acid Oxidation (map00071).

RNA-Seq Analysis

Cells were washed in PBS and lysed. Lysate was flash frozen in liquid nitrogen and stored at -80°C until the RNA was extracted at a later date. RNA extraction and purification was done using the miRNeasy Minikit (Qiagen). Purified RNA samples were sent to the University of Colorado Genomics Core Laboratory for analysis. Mapping and bioinformatics analyses were supported in part by the Biostatistics Shared Resources of Colorado's NIH/NCI Cancer Center (support grant P30CA046934). Quality control was done to trim reads to Q15 quality score, removing anything that was trimmed to less than 40 base pairs. Reads were mapped to hg19 with GSNAP, using dbSNP to guide mapping. Expression was called using Cufflinks. Genes were removed if mean reads per group (RPG or LPG) were less than 0.1 FPKM, or there were more than 50% of subjects without reads in either group.

Statistical Analysis

STRING Database 10.0 was used to analyze for RNA-Seq enrichment in genes with RPG vs LPG *P*-values <0.05. Output is a single *P*-value for protein-protein (or in this case gene-gene) interaction. Only protein coding genes were included. Go Biological Processes and KEGG pathway enrichment significance is expressed by FDR value calculated in silico by Benjamini-Hochberg, and in all cases for STRING were <0.05. Analysis was stringently limited to only those interactions with confidence values of >0.9.

Results and Discussion

Several other acylcarnitines were different between the RPG and LPG groups in relation to maternal BMI in uMSC-adipocytes. Most notably, in the OB-only infants C3DC (malonly-carnitine) was higher in the RPG group (*P* = 0.02), but in NW-only infants it was lower in the RPG group (*P* = 0.05, 2-way ANOVA interaction *P* = 0.001). Malonyl-CoA and its carnitine esther C3DC are known markers of *de novo* lipogenesis, and key in lipid metabolic regulation. Similarly, C5:1 (tiglyl-carnitine) was higher in the RPG infants born to OB mothers (*P* = 0.01), but lower in the RPG infants born to NW mothers (*P* = 0.03, 2-way ANOVA interaction *P* = 0.0008). C5OH (3-hydroxyisovaleryl-2-methyl-3-hydroxybutyryl-carnitine) was also higher in the RPG infants of OB mothers (*P* = 003, 2-way ANOVA interaction p=0.002). Both C5:1 and C5OH are known intermediates of BCAA catabolism and ketone production. Therefore, besides elevations of long chain acylcarnitines in offspring of OB mothers, related to their RPG, there were also higher short, odd chain acylcarnitines associated with both BCAA and lipid metabolism. This may indicate further repercussions on energy metabolism beyond long chain fats.

References

1 Boyle, K. E. *et al.* Mesenchymal stem cells from infants born to obese mothers exhibit greater potential for adipogenesis: The Healthy Start BabyBUMP Project. *Diabetes* **65**, 647-659 (2016).

2 Janderová, L., McNeil, M., Murrell, A. N., Mynatt, R. L. & Smith, S. R. Human mesenchymal stem cells as an in vitro model for human adipogenesis. *Obes. Res.* **11**, 65-74 (2003).

3 Gang, E. J. *et al.* Skeletal myogenic differentiation of mesenchymal stem cells isolated from human umbilical cord blood. *Stem Cells* **22**, 617-624 (2004).

Supplemental Table 1

Maternal and offspring phenotypes grouped by maternal ppBMI and subgrouped by percent fat mass.

		OB-only (n = 12)			NW-only (n = 11)		
**Group**	**Phenotype**	**RPG (n = 5)**	**LPG (n = 7)**	***P*-value (RPG/LPG)**	**RPG (n = 5)**	**LPG (n = 6)**	***P*-value (RPG/LPG)**
**Maternal**	ppBMI (kg/m^2)	**33.5±2.02**	**38.38±4.16**	**0.05 (0.87)**	20.92±0.9	20.95±1.1	0.97 (1)
	GWG (kg)	5.03±4.08	9.23±7.65	0.33 (0.55)	14.77±4.09	12.92±3.16	0.46 (1.14)
	FFA (mg/dL)	479.6±145.3	507.83±189.43	0.81 (0.94)	413.4±80.01	441.33±115.75	0.69 (0.94)
	Glucose (mg/dL)	80.8±4.26	77.33±5.37	0.32 (1.04)	81.4±14.73	78.67±5.91	0.72 (1.03)
	Insulin (mU/mL)	15.2±1.94	14.17±4.06	0.65 (1.07)	13.6±12.06	12.33±7.04	0.85 (1.1)
	Triglycerides (mg/dL)	191.25±21.55	141.33±41.03	0.08 (1.35)	132.25±26.07	155.75±31.63	0.36 (0.85)
	HOMA-IR	3.05±0.54	2.73±0.87	0.53 (1.12)	3.13±3.3	2.44±1.54	0.69 (1.28)
	Gestational age (weeks)	39.46±0.4	39.41±0.7	0.9 (1)	39.8±0.8	39.79±0.95	0.98 (1)
	Age at delivery (y)	29.71±6.4	27.35±7.02	0.6 (1.09)	31.36±3.54	29.47±5.26	0.55 (1.06)
**Infant**	Sex, n (male/female)	4/1	4/3	0.58 (NA)	1/4	5/1	0.08 (NA)
	Breastfed only, n	1/5	1/7	1 (NA)	3/5	2/6	0.57 (NA)
	Birth weight (g)	3336±163.78	3172.86±337.91	0.38 (1.05)	3294±322.39	3448.67±427.48	0.56 (0.96)
	FM – Neo (%)	**7.56±2.98**	**12.88±3.15**	**0.03 (0.59)**	10.67±1.21	7.81±3.13	0.12 (1.37)
	FFM – Neo (%)	**92.44±2.98**	**87.12±3.15**	**0.03 (1.06)**	89.33±1.21	92.19±3.13	0.12 (0.97)
	FM – Neo (g)	0.24±0.11	0.4±0.14	0.1 (0.61)	0.33±0.05	0.26±0.12	0.29 (1.27)
	FFM – Neo (g)	2.91±0.13	2.64±0.25	0.08 (1.1)	2.79±0.21	3±0.26	0.22 (0.93)
	BM – Neo (g)	3.15±0.22	3.04±0.37	0.59 (1.04)	3.13±0.24	3.27±0.35	0.52 (0.96)
	BM (delta)	3.83±0.62	3.61±0.83	0.68 (1.06)	4.13±0.52	3.55±0.64	0.18 (1.16)
	%FM (delta)	**20.34±5**	**7.95±3.92**	**2.4E-3 (2.56)**	**20.31±2.65**	**11.21±2.61**	**5.87E-4 (1.81)**
	Age, months (5mo visit)	4.34±0.4	4.91±0.85	0.23 (0.88)	4.49±0.4	4.99±0.67	0.22 (0.9)
	FM – 5mo (%)	**27.9±2.87**	**20.03±2.49**	**9.51E-4 (1.39)**	**30.98±2.84**	**19.02±1.88**	**3.52E-5 (1.63)**
	FFM – 5mo (%)	**72.1±2.87**	**79.97±2.49**	**9.51E-4 (0.9)**	**69.02±2.84**	**80.98±1.88**	**3.52E-5 (0.85)**
	FM – 5mo (g)	**1.96±0.34**	**1.36±0.25**	**9.31E-3 (1.44)**	**2.25±0.3**	**1.31±0.26**	**6.08E-4 (1.73)**
	FFM – 5mo (g)	5.02±0.3	5.42±0.76	0.33 (0.93)	5±0.27	5.51±0.54	0.12 (0.91)
	BM – 5mo (g)	6.98±0.54	6.78±0.92	0.7 (1.03)	7.26±0.45	6.82±0.76	0.33 (1.06)

Data are mean ± SD. Significance determined by Student's *t*-test.

RPG, rapid postnatal gain in adiposity; LPG, lower postnatal gain in adiposity; OB-only, infants from obese mothers; NW-only, infants from normal-weight mothers; ppBMI, prepregnancy body mass index; GWG, gestational weight gain; FFA, free fatty acids; Neo, neonate; FM, fat mass; FFM, fat-free mass; BM, body mass; %FM, percent fat mass; 5mo, 5 months of age.

Supplemental Table 2

Acylcarnitine and amino acid analyses on media from uMSC-adipocytes and uMSC-myocytes.

			uMSC-Adipocyte (*P* -value (RPG/LPG))			uMSC-Myocyte (*P* -value (RPG/LPG))		
Class	Pathway/Enzyme	Analyte	Total	OB-Only	NW-Only	Total	OB-Only	NW-Only
Acylcarnitine	Carn	C0	0.128 (0.921)	0.867 (0.983)	**0.029 (0.872)**	0.681 (1.018)	0.782 (0.983)	0.496 (1.049)
Acylcarnitine	Carn	C0/(C16+C18)	**0.022 (0.399)**	0.066 (0.346)	0.183 (0.484)	0.445 (0.733)	0.933 (1.047)	0.205 (0.453)
Acylcarnitine	Carn/LCAC	C16	**0.03 (1.757)**	**0.036 (2.596)**	0.267 (1.424)	0.059 (1.731)	0.146 (1.896)	0.221 (1.58)
Acylcarnitine	Carn/LCAC	C18:1	**0.023 (1.703)**	0.076 (2.284)	0.184 (1.426)	0.149 (1.482)	0.663 (1.208)	0.174 (1.673)
Acylcarnitine	Carn/LCAC	C18	**0.001 (1.903)**	**0.016 (2.991)**	0.059 (1.414)	0.341 (1.275)	0.559 (1.15)	0.475 (1.303)
Acylcarnitine	LCAC	C14	0.063 (1.34)	**0.02 (1.911)**	0.788 (1.05)	0.83 (1.036)	0.154 (0.671)	0.057 (1.481)
Acylcarnitine	LCOH	C18:1OH	0.141 (1.24)	0.854 (0.95)	**0.01 (1.508)**	0.397 (1.174)	0.424 (0.794)	0.067 (1.642)
Acylcarnitine	LCOH	C16:1OH	0.073 (1.324)	0.055 (1.495)	0.468 (1.2)	0.671 (0.882)	0.153 (0.474)	0.154 (1.616)
Acylcarnitine	MCAC	C8:1	**0.041 (1.457)**	**0.012 (2.086)**	0.857 (1.037)	0.86 (1.034)	0.473 (1.26)	0.611 (0.879)
Acylcarnitine	MCOH/DCAC	C3DC+C8OH	0.136 (1.266)	**0.017 (2.015)**	**0.048 (0.837)**	0.78 (0.961)	0.935 (1.017)	0.679 (0.918)
Acylcarnitine	MCOH/DCAC	C5DC+C10OH	0.175 (0.846)	0.9 (1.021)	**0.041 (0.705)**	0.26 (0.818)	0.13 (0.716)	0.597 (0.871)
Acylcarnitine	OCAC	C5:1	0.852 (1.025)	**0.012 (1.478)**	**0.033 (0.703)**	0.332 (0.876)	0.704 (0.908)	0.294 (0.853)
Acylcarnitine	OCOH	C5OH	0.352 (1.167)	**0.003 (1.837)**	0.18 (0.75)	0.326 (0.907)	0.759 (0.944)	0.222 (0.878)
Acylcarnitine	SCOH	C4OH	0.303 (1.119)	**0.012 (1.39)**	0.436 (0.9)	0.193 (0.817)	0.383 (0.784)	0.398 (0.859)
Amino Acid	1-Carbon	Threonine	0.25 (0.96)	0.655 (0.972)	0.27 (0.951)	**0.033 (0.863)**	0.172 (0.896)	0.101 (0.83)
Amino Acid	1-Carbon	Glycine	0.247 (0.855)	0.237 (0.741)	0.879 (0.982)	**0.019 (0.759)**	0.055 (0.732)	0.185 (0.78)
Amino Acid	1-Carbon	2-Aminobutyric acid	**0.0004 (0.634)**	**0.022 (0.635)**	**0.006 (0.635)**	1 (1)	0.724 (1.064)	0.699 (0.94)
Amino Acid	1-Carbon	Cysteine	**0.007 (1.705)**	0.11 (1.616)	**0.044 (1.761)**	**0.028 (1.641)**	0.14 (1.901)	0.155 (1.447)
Amino Acid	1-Carbon	Homocysteine	0.064 (2.889)	0.758 (1.324)	**0.001 (8.59)**	0.18 (0.645)	0.775 (0.904)	0.162 (0.414)
Amino Acid	1-Carbon	Met:AABU	**0.0003 (1.512)**	**0.005 (1.516)**	**0.008 (1.504)**	0.395 (0.902)	0.48 (0.864)	0.706 (0.941)
Amino Acid	1-Carbon	Met:Cys	**0.009 (0.52)**	0.134 (0.612)	0.056 (0.449)	**0.016 (0.509)**	0.178 (0.559)	0.059 (0.471)
Amino Acid	1-Carbon	Cys:AABU	**0.001 (2.67)**	**0.015 (2.474)**	**0.011 (2.762)**	**0.041 (1.752)**	0.193 (1.849)	0.169 (1.659)
Amino Acid	Arg&Pro	Ornithine	0.166 (0.77)	**0.048 (0.577)**	0.843 (0.947)	0.575 (0.892)	0.356 (0.769)	0.992 (0.997)
Amino Acid	Asp/Ala/Glu	Aspartic acid	**0.035 (0.43)**	0.066 (0.283)	0.315 (0.668)	**0.011 (0.536)**	**0.016 (0.377)**	0.295 (0.804)
Amino Acid	Asp/Ala/Glu	Asparagine	0.589 (0.84)	0.093 (0.625)	0.825 (1.145)	**0.039 (0.394)**	0.205 (0.307)	0.128 (0.45)
Amino Acid	Asp/Ala/Glu	Glutamic acid	0.423 (0.862)	0.067 (0.605)	0.642 (1.131)	0.199 (0.823)	**0.017 (0.62)**	0.91 (1.026)
Amino Acid	Asp/Ala/Glu	Alanine	0.179 (0.761)	0.337 (0.708)	0.414 (0.816)	**0.03 (0.675)**	**0.05 (0.59)**	0.347 (0.774)
Amino Acid	Histidine	1-Methylhistidine	0.862 (1.029)	0.889 (0.957)	0.683 (1.086)	**0.024 (0.794)**	0.057 (0.762)	0.238 (0.823)
Amino Acid	Histidine	Histidine	0.12 (0.943)	0.534 (0.958)	0.139 (0.93)	**0.044 (0.87)**	0.139 (0.893)	0.135 (0.844)
Amino Acid	Lys&Trp	2-Aminoadipic acid	0.116 (0.373)	0.137 (0.239)	0.606 (0.692)	**0.048 (0.25)**	0.184 (0.241)	0.196 (0.274)
Amino Acid	Phe&Tyr	Tyrosine	0.227 (0.958)	0.525 (0.959)	0.325 (0.958)	**0.035 (0.865)**	0.14 (0.893)	0.114 (0.835)
Amino Acid	Phe&Tyr	Phenylalanine	0.275 (0.961)	0.588 (0.964)	0.349 (0.96)	**0.034 (0.865)**	0.143 (0.898)	0.108 (0.832)
Amino Acid	Total/Phe&Tyr	Total AAA	0.251 (0.96)	0.557 (0.961)	0.336 (0.959)	**0.034 (0.865)**	0.141 (0.896)	0.111 (0.834)
Amino Acid	Total	Total Non-Essential	0.139 (0.914)	0.219 (0.851)	0.524 (0.969)	**0.022 (0.835)**	**0.012 (0.821)**	0.214 (0.842)

Significance determined by Student's t-test.

RPG, rapid postnatal gain in adiposity; LPG, lower postnatal gain in adiposity; OB-only, infants from obese mothers; NW-only, infants from normal-weight mothers.

Supplemental Table 3

RNA-Seq analysis on uMSC-myocytes.

Grouping	Nodes	Edges	*P*-value	Notes	Biologic Process	Examples (FDR)	KEGG	Examples (FDR)
Total cohort	1082	855	0	NA	72	Nitrogen Compound Metabolic Processing (3.7E-10)<br>RNA Processing (1.42E-09)<br>Gene Expression (1.55E-08)	1	Spliceosome (6E-04)
Total cohort (positive only)	260	32	3.69E-08	Includes MYLK, MRAS, and CTSB	4	Muscle Organ Development (0.03)<br>Muscle Structure Development (0.03)	2	Lysosome (0.04)
Total cohort (negative only)	781	651	0	Includes EP300-CREBBP-HDAC1	95	RNA Metabolic Process (2.8E-15)<br>Nitrogen Compound Metabolic Processing (1.9E-14)<br>Gene Expression (3.98E-13)<br>Cellular Biosynthetic Process (7.32E-06)<br>Mitotic Cell Cycle (1.5E-03)	1	Spliceosome (1.39E-05)
OB-only	1432	1344	0	Includes EP300-CREBBP-HDAC1	119	Cellular Metabolic Process (1.97E-15)<br>Biosynthetic Process (4.86E-10)<br>Gene Expression (4.21E-08)<br>RNA Metabolic Process (3.61E-06)<br>Apoptosis (0.01)	0	NA
OB-only (positive only)	791	438	0	NA	68	Protein Metabolic Process (5.38E-08)<br>Mitochondrion Organization (3.83E-03)<br>Organic Acid/Oxoacid Metabolic Process (4.98E-03)<br>Apopotic Process (0.01)<br>Response to Unfolded Protein (0.02)<br>Oxidation-Reduction Process (0.03)	15	Metabolic Pathways (1.23E-05)<br>Lysosome (1.18E-04)<br>Oxidative Phosphorylation (3.98E-03)<br>NAFLD (3.98E-03)<br>Proteosome (9.77E-03)<br>Sulfur Metabolism (0.01)<br>Protein Export (0.01)
OB-only (negative only)	642	382	0	Includes EP300-CREBBP-HDAC7	112	Nucleic Acid Metabolic Process (2E-26)<br>Gene Expression (1.73E-22)<br>Cellular Nitrogen Compound Metabolic Process (1.15E-21)<br>Histone Modification (1.19E-08)	0	NA
NW-only	646	304	0		34	Nitrogen Compound Metabolic Processing (3.5E-04)<br>RNA Processing (0.02)<br>Mitochondrion Organization (0.03)<br>Gene Expression (0.05)	0	NA
NW-only (positive only)	163	10	0.17	NA	6	NA	0	NA
NW-only (negative only)	483	220	0		35	Nitrogen Compound Metabolic Processing (4.46E-04)<br>Mitochondrion Organization (7.11E-04)<br>RNA Processing (3.17E-03)<br>Mitochondrial Translation (5.87E-03)<br>Protein Metabolic Processes (0.02)	1	Peroxisome (0.03)

OB-only, infants from obese mothers; NW-only, infants from normal-weight mothers; FDR, false discovery rate.

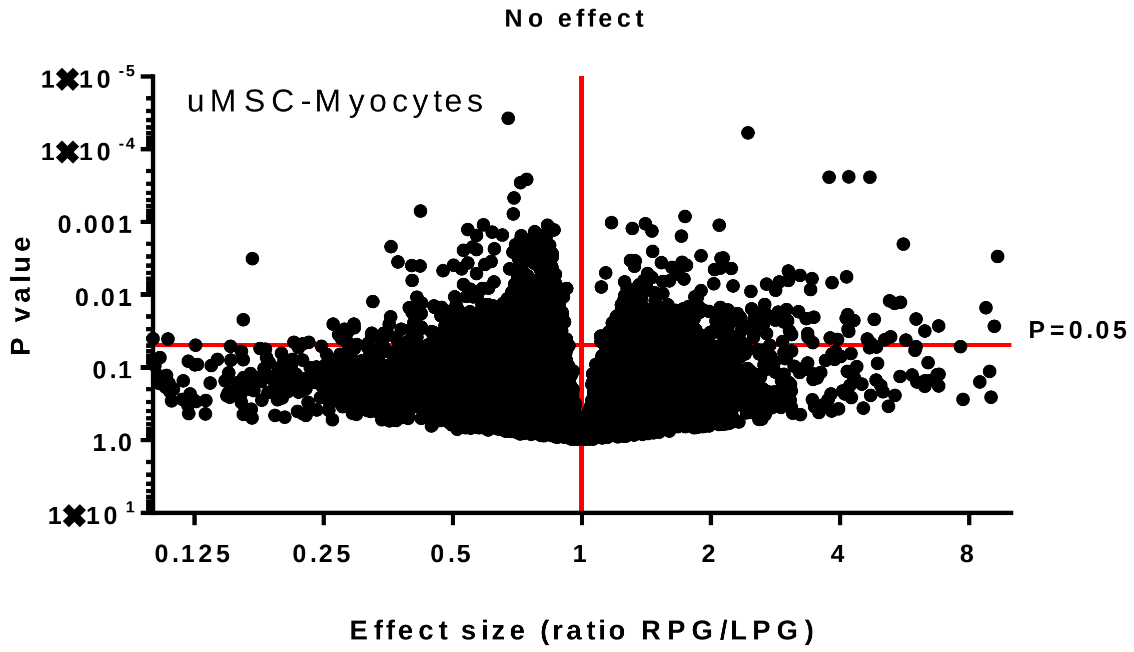

Supplemental Figure 1: Volcano plot of differential gene expression (RPG/LPG) in uMSC-myocytes. In total, 1,080 protein coding genes were significantly differentially expressed between the two groups.

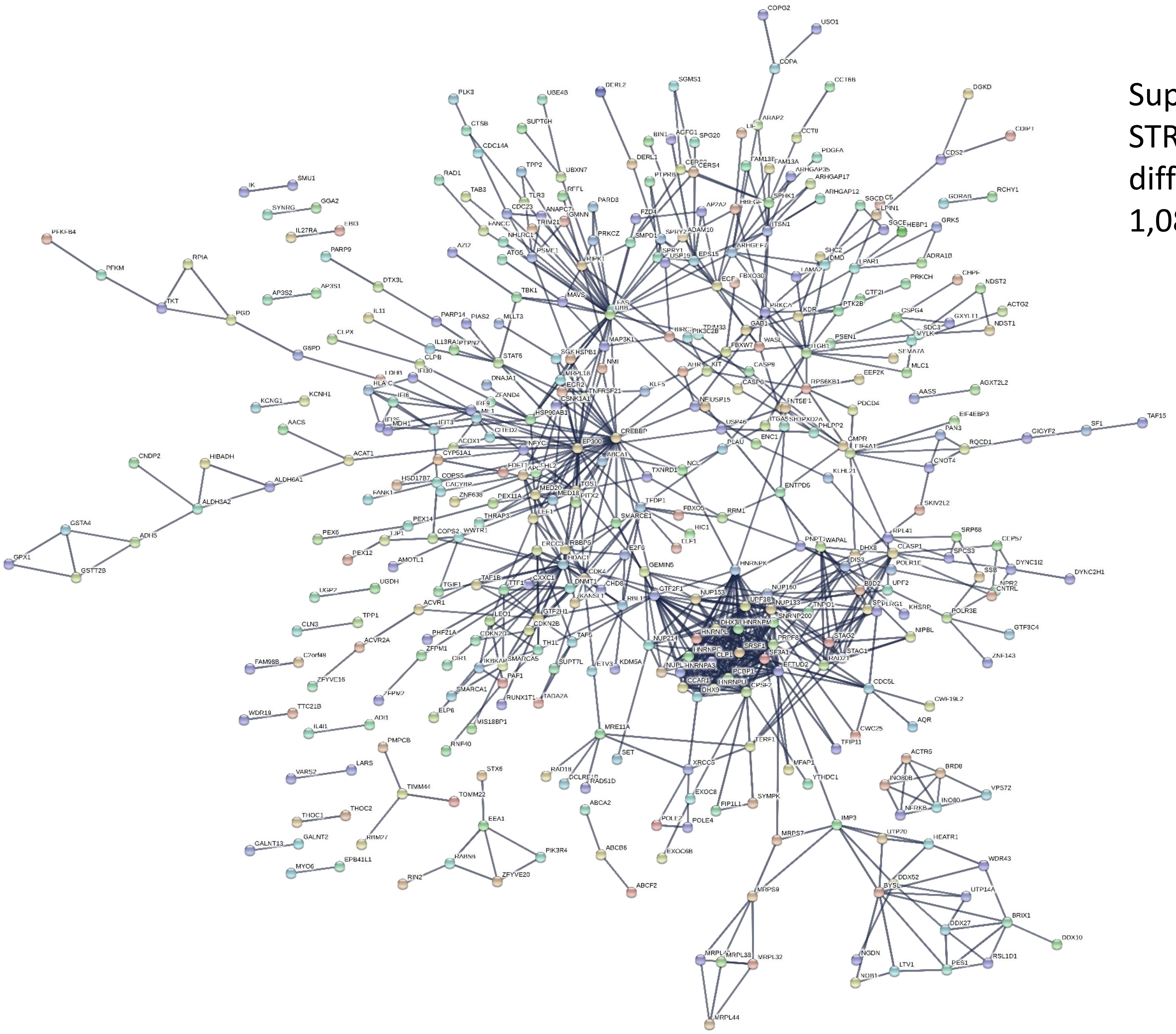

Supplemental Figure 2: Network diagram for STRING enrichment analysis in significantly differentially expressed genes (RPG/LPG, n = 1,080) in uMSC-myocytes.
